# Expert consensus on the use of trazodone in patients with major depressive disorder: results from a European Delphi panel

**DOI:** 10.1192/j.eurpsy.2026.10162

**Published:** 2026-02-06

**Authors:** Allan Hunter Young, Andrea Fagiolini, Luis Agüera-Ortiz, Marcin Siwek, Siegfried Kasper

**Affiliations:** 1 Psychological Medicine, King’s College London, London, UK; 2 Faculty of Medicine and Surgery, University of Siena, Italy; 3 Department of Psychiatry, Complutense University of Madrid, Madrid, Spain; 4 Department of Biological and Community Psychiatry, Jagiellonian University Medical College, Krakow, Poland; 5 Department of Psychiatry and Psychotherapy, Clinical Division of General Psychiatry, Medical University of Vienna, Vienna, Austria

**Keywords:** Major depressive disorder, trazedone, serotonin antagonist and reuptake inhibitor, antidepressants, delphi study, patient profile

## Abstract

**Background:**

Trazodone is commonly used in the treatment of major depressive disorder (MDD) in adults. This study aimed to establish consensus on the clinical scenarios and patient profiles for which trazodone treatment is considered suitable.

**Methods:**

A two-round Delphi process was conducted across eight European countries. Statements regarding trazodone were rated by a panel of 32 experts for agreement or disagreement using a 9-point Likert scale; those with <70% agreement among panelists were revised and reassessed by the panel.

**Results:**

There was strong consensus agreement on 68 out of 91 statements (75%) related to trazodone. According to the panel, trazodone is well tolerated, with low anticholinergic activity, minimal impact on sexual function, weight neutrality, and low potential for clinically relevant drug–drug interactions. Consensus agreement supported trazodone use across a broad spectrum of patients with MDD, including those with insomnia, anxiety, psychomotor agitation, substance use, physical comorbidities, neurological conditions, and treatment-resistant depression; consensus agreement was also achieved for trazodone use in elderly patients, and those experiencing adverse effects with other antidepressants.

**Conclusions:**

This study suggests that trazodone is useful in the treatment of MDD across multiple patient profiles. These findings offer practical guidance to support individualized and evidence-based decision-making in clinical practice.

## Introduction

Major depressive disorder (MDD) is the most prevalent mood disorder and is expected to rank first in terms of global disease burden by 2030, according to the World Health Organization [1]. It is currently the largest contributor to disability worldwide [2], impacting multiple domains including daily living, productivity, and quality of life (QoL).

Antidepressants are the most common treatment for MDD. These agents comprise many different classes, with different mechanisms of action and safety/tolerability profiles. However, many patients fail to achieve long-term remission with antidepressants, often due to suboptimal treatment choices, adverse effects, or poor adherence to medication [3, 4].

Trazodone, discovered in the 1960s, is a serotonin antagonist and reuptake inhibitor (SARI) authorized for the treatment of depressive disorders in adults, including those associated with symptoms of anxiety, and is still commonly used in the treatment of MDD [5, 6]. Through its multimodal and multifunctional mechanism of action (MoA), trazodone supports efficacy across core symptoms and associated features, such as sleep disturbances and anxiety [5, 7], while maintaining a favorable safety profile [8]. However, despite its long-standing availability and widespread use in clinical practice, the positioning of trazodone in the treatment of MDD remains heterogeneous. Trazodone is prescribed across a broad spectrum of doses and formulations and is frequently selected for patients with complex clinical profiles, including comorbid insomnia, anxiety symptoms, medical or neurological comorbidities, older age, or intolerance to other antidepressants. However, existing clinical guidelines often provide limited or non-specific recommendations regarding these real-world scenarios. Moreover, the available evidence base, while substantial, is fragmented across different indications, dose ranges, and study designs, making it difficult for clinicians to translate trial data into individualized treatment decisions. As a result, prescribing practices may vary considerably across countries and clinical settings, potentially reflecting a gap between evidence, expert knowledge, and routine care.

To this end, a structured Delphi consensus among experienced clinicians was considered an appropriate methodology to systematically capture expert judgment, identify areas of agreement, and provide practical guidance where empirical data alone may be insufficient. This Delphi study aimed to establish consensus on key features of trazodone in the treatment of MDD, including MoA, efficacy, safety/tolerability, drug–drug interactions, and patient profiles for which trazodone is considered most suitable.

## Methods

### Study design

This was a two-round, modified Delphi process conducted across eight European countries. The study was coordinated by a Scientific Committee (SC) who developed a questionnaire regarding trazodone use in clinical practice based on a literature review and clinical experience. Questionnaires were completed by a panel of invited psychiatrists and neurologists. As this was a non-interventional, expert consensus with no patient data, no Ethics Committee approval was required.

### Participants and roles

The SC, comprising five psychiatrists with expertise in MDD and trazodone, was responsible for validating the study design and protocol, defining the inclusion criteria for panelists, reviewing the results of the consensus, and providing clinical oversight throughout the process. A panel of 32 invited clinicians, including 27 psychiatrists and 5 neurologists, was recruited for the Delphi process. Inclusion criteria for the panelists included: professionals from psychiatry and neurology fields; practicing in one of eight European countries (Austria, Bulgaria, Italy, Poland, Portugal, Romania, Spain, and Turkey); ≥5 years of experience in diagnosing and treating MDD as an isolated condition or in the context of other morbidities; and regular clinical use of trazodone, defined as treating more than 10 patients per month. The SC was invited to suggest panelists from their country of origin and these suggestions were considered when feasible and in line with data protection regulations. Neurologists were included if their expertise was relevant to the consensus objectives (e.g., in relation to trazodone use in mild depression or specific clinical manifestations such as insomnia).

### Literature review and structured questionnaire

The literature search was conducted in PubMed using the following criteria: English-language articles including the terms trazodone (or commercial names), MDD, and either patient profiles, drivers, barriers, or prescriptions; studies on animals, children, and non-Caucasian populations were excluded. A total of 186 publications were identified and 82 abstracts were retained for in-depth analysis as they addressed the research questions and satisfied the inclusion and exclusion criteria (Supplementary Figure 1). Information from a narrative review of 67 full papers was collected using Microsoft Excel and used to identify key clinical themes and evidence gaps related to trazodone use. The SC then developed a structured 28-item questionnaire, comprising 20 items on panelists’ profiles and MDD management practices, and 8 items (including 91 statements) on trazodone mechanism of action, efficacy, tolerability, safety, drug–drug interactions, and appropriate patient profiles.

### Delphi process

The structured questionnaire was provided to each panelist. Panelists responded to each question, rating their agreement/disagreement with statements using a 9-point Likert scale (1–3 conveyed disagreement with the statement; 4–6 conveyed neither agreement nor disagreement; 7–9 conveyed agreement). Consensus ‘agreement’ was defined as strong if ≥70% of participants agreed with a statement and moderate if ≥60–<70% of participants agreed; non-consensus was defined as <60% of participants agreeing with the statement. Similar thresholds were used to define consensus ‘disagreement’ and consensus ‘neither agreement nor disagreement’. Statements that did not reach strong consensus in the first round were revised by the SC and reassessed in the second round to allow re-evaluation and promote refinement of expert judgment.

### Statistical analysis

While consensus was defined based on predefined percentage thresholds, measures of central tendency were calculated to assess the distribution and variability of responses. Median score, interquartile range (IQR), and coefficient of variation (CV) were calculated for all items evaluated on the Likert scale. The collected data were entered into a centralized database and analyzed using IBM SPSS Statistics 27. Corresponding graphs were generated using Microsoft Excel. High variability in the data for each item was defined as a CV greater than 30%.

## Results

### Characteristics of Delphi panelists

Panelists’ demographics and experience are shown in Supplementary Table 1. All panelists were affiliated with metropolitan hospitals and/or universities. Most panelists (66%) had more than 15 years’ specialty experience and 81% had more than 15 years’ experience in MDD; 97% and 91% of panelists had experience in cognitive impairment and chronic pain, respectively. Most panelists were either Chief of Unit (34%) or Department Head (22%). Psychiatrists diagnosed/treated a median of 100 patients per month, including 50 with MDD and 15 with newly diagnosed MDD; neurologists diagnosed/treated a median of 100 patients per month, including a median of 30 with MDD and a median of 4 with newly diagnosed MDD. The majority of panelists were involved in all stages of the patient journey.

### Delphi panelists’ treatment practices

**Figure 1.**
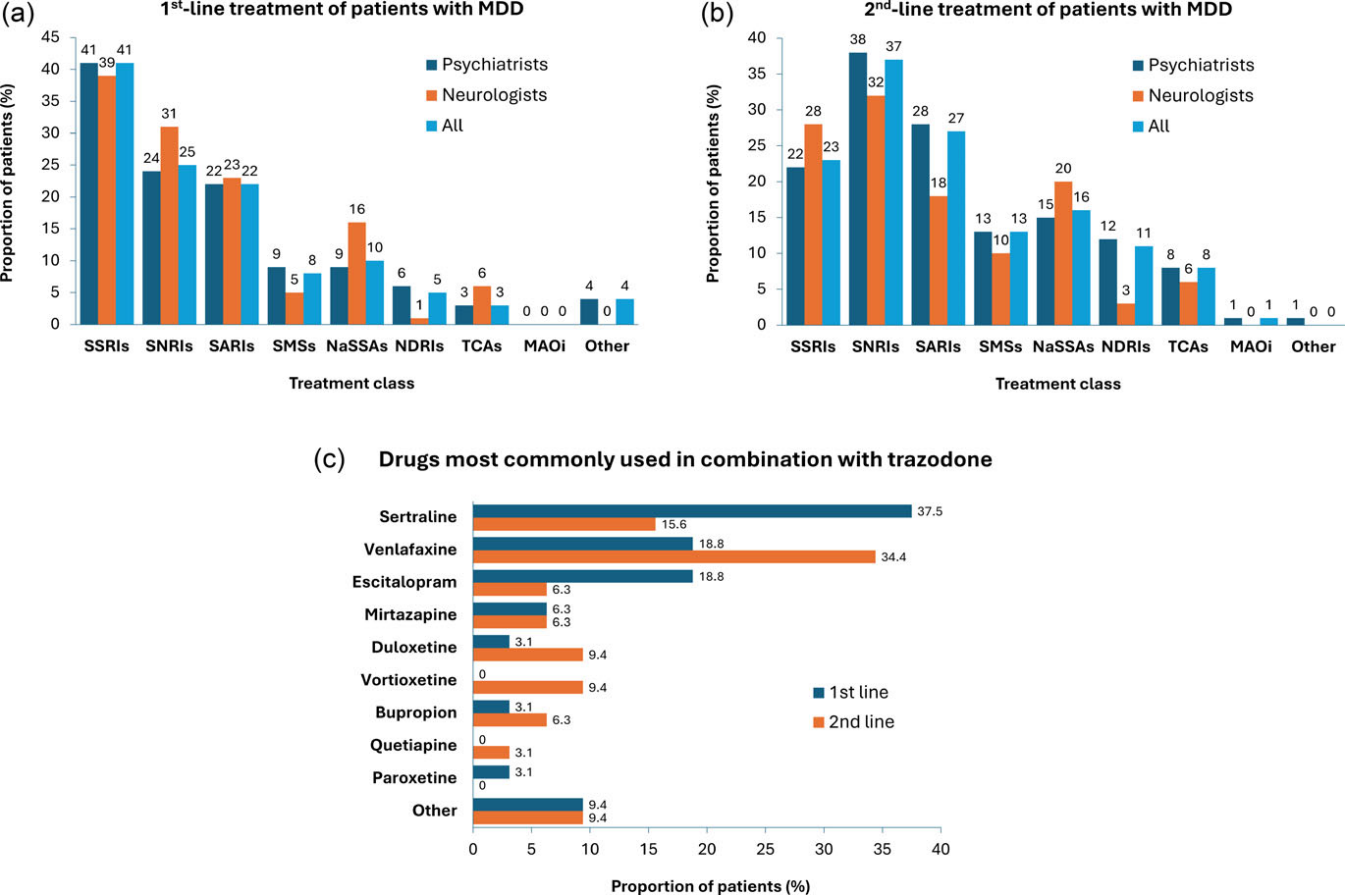
Treatments used by expert panelists in patients with MDD in (A) first line, (B) second line, and (C) in combination with trazodone. *Note:* MAOis, monoamine oxidase inhibitors; MDD, major depressive disorder; NaSSAs, noradrenergic and specific serotonergic antidepressants; NDRIs, norepinephrine/dopamine reuptake inhibitors; SARIs, serotonin antagonist and reuptake inhibitors; SMSs, serotonin modulator and stimulators; SNRIs, serotonin and norepinephrine reuptake inhibitors; SSRIs, selective serotonin reuptake inhibitor; TCAs, tricyclic antidepressants.

The most common first-line treatment classes used by the Delphi panelists in patients with MDD were selective serotonin reuptake inhibitors (SSRIs; 41% of patients), serotonin and norepinephrine reuptake inhibitors (SNRIs; 25%), and SARIs (22%); in the second line, the most common treatments were SNRIs (37%), SARIs (27%), and SSRIs (23%) (Figure 1A,B). These results were similar whether patients were treated by psychiatrists or neurologists.

According to panelists, 51% of their patients with MDD (49% for psychiatrists; 73% for neurologists) were receiving trazodone in a regular month. The most common formulations used in patients treated with trazodone were prolonged-release tablets (45%; 42% for psychiatrists; 67% for neurologists), extended-release Contramid® tablets (27%; 31% for psychiatrists; 0% for neurologists), immediate-release tablets (25%; 24% for psychiatrists; 33% for neurologists), oral drops (2%; Italy only – not available in all European countries) and injectable liquid solution (1%; Italy only). Availability of trazodone formulations varies across European countries, which may have influenced panelists’ prescribing patterns; for example, prolonged-release tablets are the only formulation available in Austria and immediate-release tablets are the only formulation available in Spain.

For patients treated with trazodone in the first line, 34% received trazodone monotherapy and 66% received trazodone as combination therapy according to panelists; in the second line, 19% received trazodone as monotherapy and 81% as combination therapy. These results were similar whether reported by psychiatrists or neurologists. The most common drugs used in combination with trazodone by the psychiatrists and neurologists were sertraline and venlafaxine, respectively (Figure 1C).

Psychiatrists were more likely to use trazodone at a higher dosage than neurologists; 55% of psychiatrists used trazodone at a high dose (≥150 mg/day) compared with 31% of neurologists. Panelists reported that most patients treated with trazodone monotherapy received a high dose in the first (56%) and second (69%) lines; in contrast, most patients receiving combination therapy received a low dose in the first (72%) and second (66%) lines. This pattern was similar whether reported by psychiatrists or neurologists.

Discontinuation rates for trazodone were 12% overall (12% reported by psychiatrists; 11% reported by neurologists). Reasons for discontinuation were tolerability issues (37%), patient preference (27%), lack of efficacy (23%), safety concerns (4%), limited formulation availability (3%), interaction with other drugs (2%), and other causes (5%).

Panelists reported that most patients (66%) were receiving trazodone for at least 7 months (5% for <1 month; 9% for 1–3 months; 20% for 4–6 months; 28% for 7–12 months; 22% for 13–24 months; and 16% for >2 years).

### Delphi panel consensus statements

In the modified Delphi survey, strong consensus agreement (i.e., agreement among ≥70% of panelists) was achieved for 68 of 91 statements (75%) related to trazodone, including 7 statements on MoA (Table 1), 10 on efficacy (Table 2), 16 on safety/tolerability (Table 3), 5 on drug–drug interactions (Table 4), and 30 on patient profiles (Table 5). Strong consensus agreement was achieved for 49 statements in Round 1 and an additional 19 statements in Round 2.

**Table 1.** Mechanism-of-action statements achieving consensus agreement

Statement	Median (IQR) score	Agreement (% panelists)
*Strong consensus of agreement (i.e., by ≥ 70% of participants)*		
At high doses (>150 mg) trazodone exerts its action blocking SERT	9 (1)	100
Trazodone multimodal MoA addresses mood symptoms and sleep issues effectively	8 (1)	100
Trazodone is a multifunctional and multimodal drug with dose-dependent pharmacological actions	9 (1)	100
At low doses (<150 mg) trazodone exerts its action on 5-HT2A receptors, α1 adrenergic receptors, and H1 receptors	9 (1)	97
Trazodone’s multimodal MoA addresses mood symptoms and anxiety effectively	8 (2)	91
At high doses (>150 mg), trazodone’s affinity for 5-HT2C and 5-HT7 receptors is comparable to its affinity for SERT	8 (2)	87
Trazodone’s partial agonist actions at the 5-HT1A receptors, combined with its SERT inhibition, as well as its antagonism of 5-HT2C and 5-HT7 receptors, may contribute to its faster onset of antidepressant action and enhanced efficacy in treating MDD	7 (2)	81

Abbreviations: 5-HT, 5-hydroxytryptamine; IQR, interquartile range; MDD, major depressive disorder; MoA, mechanism of action; SERT, serotonin transporter.

**Table 2.** Efficacy statements achieving consensus agreement

Statement: In adult MDD patients, trazodone…	Median (IQR) score	Agreement (% panelists)
*Strong consensus of agreement (i.e., by ≥ 70% of participants)*		
…controls symptoms of insomnia and improves overall sleep quality	9 (1)	100
…demonstrates a rapid onset of antidepressant action, particularly in alleviating depressive symptoms such as insomnia and psychomotor agitation	8 (1)	97^a^
…improves depressive symptoms, which may positively impact cognition	8 (1)	91^a^
…is an adequate treatment for anxiety symptoms	8 (1.5)	88
…maintains its efficacy over the long-term	8 (2)	88
…is an efficacious/effective drug for the treatment of patients with MDD	8 (2)	88
…is effective in reducing depressive symptoms in patients with comorbid chronic pain	8 (1)	88^a^
…is effective in reducing the craving and may allow for a gradual discontinuation of benzodiazepines in people addicted to benzodiazepine	8 (1.25)	88^a^
…is effective in alleviating depressive symptoms associated with cannabis, cocaine or alcohol addiction like insomnia, agitation, or depressed mood	8 (2)	81^a^
…is effective in reducing depressive symptoms in patients with comorbid neuropathic pain	7.5 (1)	78^a^

Abbreviations: IQR, interquartile range; MDD, major depressive disorder.

a Consensus achieved in Round 2 of Delphi process.

**Table 3.** Safety and tolerability statements achieving consensus agreement

Statement: In adult MDD patients, trazodone…	Median (IQR) score	Agreement (% panelists)
*Strong consensus of agreement (i.e., by ≥ 70% of participants)*		
… has a good safety profile and therefore can be used long-term	8 (1)	100
… has minimal impact on sexual dysfunction	8 (1)	100
… may provide sedative effects beneficial for managing sleep disturbances	9 (1)	97
… is well-tolerated by most patients	8 (1)	94
… is not diabetogenic	8 (1)	94
… is safe as an alternative treatment for avoiding SSRI-induced sexual dysfunction (impotence)	8 (1)	90
… can be safely used in elderly patients due to its low anticholinergic properties	8 (2)	88
… orthostatic hypotensive effect is in most of the cases not clinically relevant	7 (1)	88
… has a minimal impact on motor function	7.5 (1)	84
… has a rare potential for hepatotoxicity. Periodic monitoring of liver function may be considered in patients with liver impairment, particularly in severe cases	8 (1)	84
… has minimal gastrointestinal side effects	8 (1.5)	84
… is weight neutral	7.5 (2)	81
… when combined with other medications that are known to prolong the QT interval or increase trazodone concentration, careful consideration is advised	8 (1.5)	81
… effect on cardiac repolarization (QTc) is unlikely to represent a clinical risk for ventricular arrhythmia	8 (1)	81
… shows fewer side effects than other antidepressants	7 (1.5)	75
… does not require dose adjustment in patients with renal impairment	7 (1.5)	75
*Moderate consensus of agreement (i.e., by ≥ 60–<70% of participants)*		
Caution should be exercised when prescribing trazodone to pregnant women due to the limited data available, despite the long-term availability of the drug	7 (2)	65^a^

Abbreviations: IQR, interquartile range; MDD, major depressive disorder; SSRI, selective serotonin reuptake inhibitors.

a Consensus achieved in Round 2 of Delphi process.

**Table 4.** Drug–drug interaction statements achieving consensus agreement

Statement: In adult MDD patients, trazodone…	Median (IQR) score	Agreement (% panelists)
*Strong consensus of agreement (i.e., by ≥ 70% of participants)*		
…when used concomitantly with alcohol, the risk of hypersedation may increase	8 (2)	84
…serotonin syndrome may occur when used with SSRIs, but it is very unlikely	8 (2)	81^a^
…has a low risk of drug-to-drug interactions	7.5 (1)	78
…when used concomitantly with central nervous system depressants, the risk of hypersedation may increase	8 (1.5)	75
…is a preferred antidepressant for patients receiving polypharmacy due to its low drug-to-drug interactions	7 (1.5)	75

Abbreviations: IQR, interquartile range; MDD, major depressive disorder; SSRI, selective serotonin reuptake inhibitors.

a Consensus achieved in Round 2 of Delphi process.

**Table 5.** Statements on suitable patient profiles achieving consensus agreement

Statement: Trazodone is suitable for patients with MDD…	Median (IQR) score	Agreement (% panelists)
*Strong consensus of agreement (i.e., by ≥ 70% of participants)*		
… and sleep disturbances	9 (0)	100
… and anxiety	8 (1)	94
… and agitation	8 (2)	91
… and alcohol use disorders	8 (2)	91^a^
… associated with physical comorbidities	8 (1)	91^a^
… and who are elderly and present with agitation	8 (2)	88
… and withdrawal therapy for benzodiazepine addiction	8 (2)	88
… and depressive symptoms associated with substance abuse (dual pathology)	8 (2)	88^a^
… and sexual dysfunction (impotence)	8 (2)	87
… and diabetes mellitus	7 (1)	84
… and GAD	8 (1)	84
… and who are elderly	8 (1.5)	84
… and depressive symptoms associated with chronic pain	8 (1)	84^a^
… and depressive symptoms associated with neuropathic pain	8 (1)	84^a^
… and dementia	8 (1)	81
… and MCI	7 (1)	81
… and worrying about weight gain	8 (1)	81^a^
… and Alzheimer’s disease	8 (1)	78
… and adverse effects due to other antidepressants	8 (1.5)	78
… and post-stroke depressive symptoms	7.5 (2)	78^a^
… associated with menopause	7 (1.25)	78^a^
… and treatment-resistant	7 (1.5)	75
… and Parkinson’s disease	8 (2)	74
… and epilepsy, as it has a low impact on seizure threshold, similar to SSRIs	8 (1.5)	74^a^
… associated with cancer	8 (1.5)	74^a^
… and withdrawal syndromes from substance abuse	8 (2)	72
… and cognitive deficits	7 (2)	72
… in polymedicated patient	7 (2)	72^a^
… and mild to moderate liver dysfunction, but should be used with caution in patients with severe hepatic impairment	7.5 (2)	72^a^
… and multiple sclerosis	7 (2)	70
*Moderate consensus of agreement (i.e., by ≥ 60–<70% of participants)*		
… and PTSD	8 (2)	69^a^
… and young adults (≥18 years old)	7 (2)	69^a^
… and associated with migraine	7 (2)	68^a^
… and hypertension	7 (2)	66^a^
… and bipolar disorder	7 (2)	63^a^
… and emotional blunting	7 (2)	63^a^

Abbreviations: GAD, generalized anxiety disorder; IQR, interquartile range; MCI, mild cognitive impairment; MDD, major depressive disorder; PTSD, post-traumatic stress disorder; SSRIs, selective serotonin reuptake inhibitors.

a Round 2 of Delphi process.

Statements on trazodone MoA with the highest proportion of panelists expressing agreement were: “*At high doses (>150 mg) trazodone exerts its action blocking SERT*” (100%); “*Trazodone multimodal MoA addresses mood symptoms and sleep issues effectively*” (100%); and “*Trazodone is a multifunctional and multimodal drug with dose-dependent pharmacological actions*” (100%; Table 1).

Regarding trazodone efficacy in adult patients with MDD, statements with the highest agreement among panelists included: “*…controls symptoms of insomnia and improves overall sleep quality*” (100%); *…demonstrates a rapid onset of antidepressant action, particularly in alleviating depressive symptoms such as insomnia and psychomotor agitation”* (97%); and “*…improves depressive symptoms, which may positively impact cognition*” (91%; Table 2).

Statements on trazodone safety/tolerability with the highest proportion of panelists expressing agreement were: “*…has a good safety profile and therefore can be used long-term*” (100%); “*…has minimal impact on sexual dysfunction*” (100%); and “*…may provide sedative effects beneficial for managing sleep disturbances*” (97%; Table 3).

Regarding trazodone drug–drug interactions in adult patients with MDD, statements with the highest level of agreement among panelists were: “*…when used concomitantly with alcohol, the risk of hyper sedation may increase*” (84%); “…*serotonin syndrome may occur when used with SSRIs, but it is very unlikely*” (81%); and “*…has low risk of drug-to-drug interactions*” (78%; Table 4).

Of the 30 MDD patient profiles that panelists agreed were suitable for treatment with trazodone, those with the highest consensus of agreement included patients with sleep disturbances (100%), anxiety (94%), agitation (91%), alcohol use disorders (91%), and MDD associated with physical comorbidities (91%; Table 5).

Moderate consensus of agreement (i.e., agreement among ≥60–<70% of panelists) was achieved for a further seven statements, including one on safety (Table 4) and 6 on patient profiles (Table 5). MDD patient profiles with moderate consensus of agreement for suitability of trazodone included young adults, those with PTSD, hypertension, bipolar disorder, or emotional blunting, and those with MDD associated with migraine (Table 5). Two MDD patient profiles had a strong consensus of ‘neutral’ regarding the benefit of trazodone (i.e., ≥70% of panelists expressed neither agreement nor disagreement), including patients with QT prolongation (77%) and patients with arrhythmia (74%).

No consensus was reached for 14 statements, including: whether trazodone is effective in helping with comorbid obsession; whether trazodone is effective in addressing hypersomnia; whether clinical practice suggests possible use in breastfeeding if the benefits of breast-feeding for the infant and the benefits of trazodone for the woman are positive; whether trazodone should be used in patients with MDD associated with eating disorders; and whether trazodone should be used in patients with MDD and anhedonia symptoms, atrial fibrillation, atypical depression subtype, chronic kidney disease, hypersomnia, obsessive-compulsive disorder, orthostatic hypotension, psychomotor retardation, psychosis, or risk of priapism.

**Figure 2.**
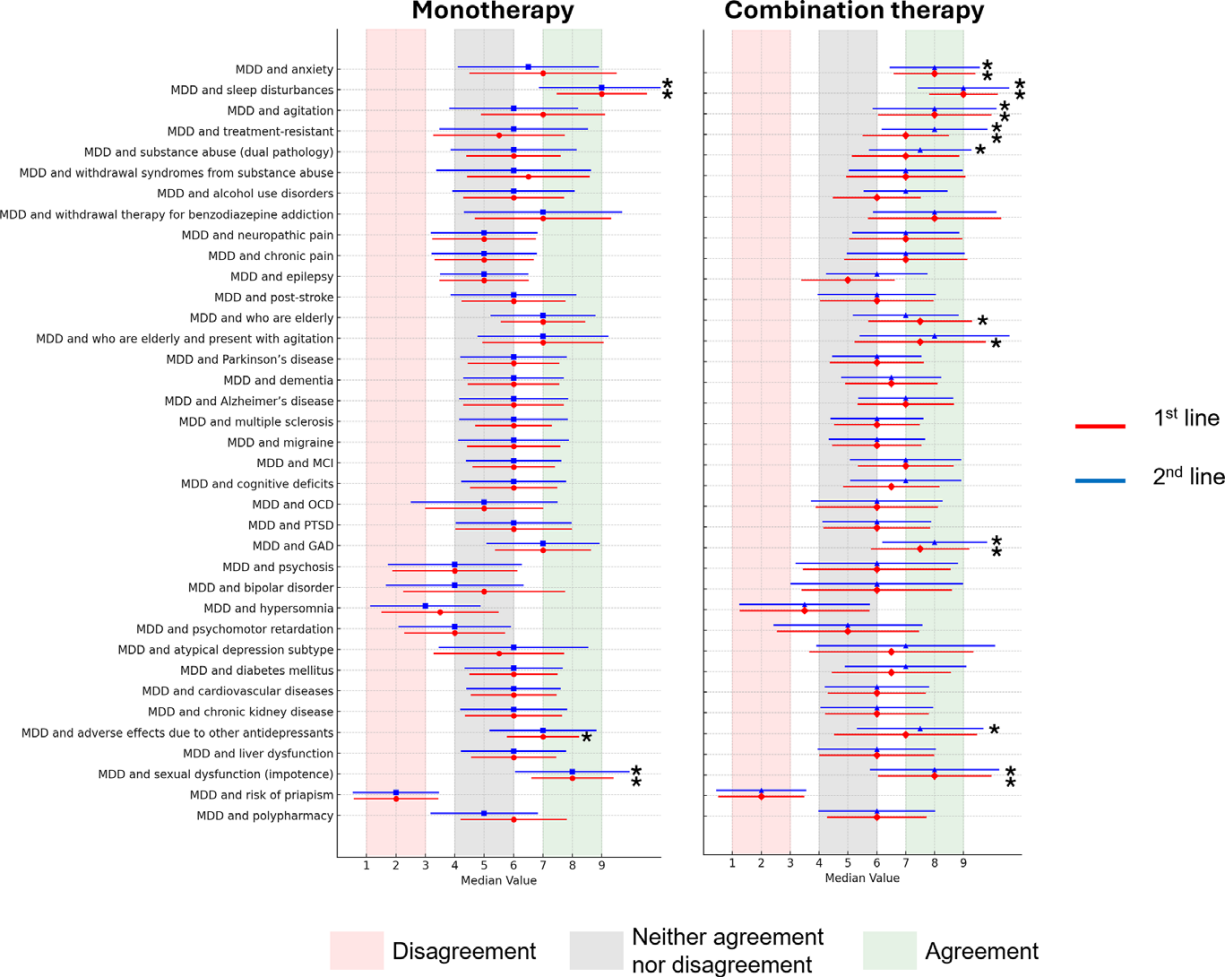
Median scores for agreement/disagreement on use of trazodone in different settings (1st and 2nd line; monotherapy and combination therapy) for specific patient profiles. Bars represent the coefficient of variation for each patient profile. *Note:* *Statements with strong consensus of agreement. GAD, generalized anxiety disorder; MCI, mild cognitive impairment; MDD, major depressive disorder; OCD, obsessive-compulsive disorder; PTSD, post-traumatic stress disorder.

The pattern of differences in median scores for agreement/disagreement across different patient profiles was similar for trazodone monotherapy and combination therapy, and for use of trazodone in the first and second lines, although median scores tended to be higher for trazodone use as combination therapy versus monotherapy (Figure 2).

## Discussion

This study presents expert consensus on the clinical application of trazodone in MDD, highlighting patient profiles that may benefit from its use. The Delphi panelists contributing to this consensus had substantial experience in the treatment of patients with MDD, including patients with cognitive impairments, chronic pain, withdrawal from substance abuse, and neuropathic pain.

The panelists reached consensus on all statements regarding trazodone’s mechanism of action, consistent with its well-established profile as a multifunctional and multimodal drug with dose-dependent pharmacological effects [7].

Consensus agreement on most efficacy statements supports trazodone’s rapid and sustained antidepressant effects as well as its benefits in improving sleep disturbances and symptoms of anxiety. There was no consensus on the effectiveness of trazodone in addressing comorbid obsession, although some evidence from case series suggests a beneficial effect in obsessive compulsive disorder (OCD) [9]. Similarly, there was no consensus on the effectiveness of trazodone in reducing hypersomnia; this is unsurprising as trazodone is typically associated with sedative properties [10], and all panelists agreed that trazodone controls symptoms of insomnia and improves overall sleep quality.

The consensus that trazodone is generally well tolerated in patients with MDD reflects trazodone’s established safety and tolerability profile [5]. In comparison with SSRIs and SNRIs, trazodone has a low risk for anxiety, insomnia, and sexual dysfunction due to its pharmacological profile (i.e., simultaneous inhibition of 5-HT2A and 5-HT2C receptors, serotonin transporters, and alpha adrenergic and histaminergic receptors) [11, 12]. There was moderate consensus on the need for caution when prescribing trazodone to pregnant women. Across the long history of trazodone use, there are no reported data to suggest a negative impact in pregnancy, although evidence is limited and largely based on case reports. One study suggests that maternal exposure to trazodone is not associated with adverse outcomes in early pregnancy [13], although it is known that trazodone is transferred to the infant during lactation [14]. Further studies are required to support safety during pregnancy and lactation, and caution should always be taken when prescribing medications during this period. As there is limited information on trazodone excretion into human breast milk, the label advises weighing the benefits of breastfeeding for the child against the benefits of trazodone treatment for the mother when deciding whether to continue or discontinue breastfeeding or trazodone therapy [6]. Consistent with this approach, a safety scoring system for the use of psychotropic drugs during lactation indicates that use of trazodone is considered “possible with caution” [15].

There was strong consensus of agreement among the panelists that trazodone has a low risk of drug–drug interactions, and as a consequence is a preferred antidepressant for patients receiving polypharmacy. However, there was also consensus that trazodone may increase the risk of hypersedation when used concomitantly with alcohol or with central nervous system depressants. The expert panelists agreed that, in their clinical practice, they have not observed cases of serotonin syndrome with trazodone, although the risk cannot be completely excluded due to its serotonergic activity.

Strong consensus agreement supported trazodone use across a broad spectrum of patients with MDD, including those with insomnia, anxiety, psychomotor agitation, coexisting substance use, physical comorbidities, neurological conditions (except migraine), menopause, cancer, alcohol use disorders, and treatment-resistant depression; consensus agreement was also achieved for trazodone use in elderly patients, those worrying about weight gain, and those experiencing adverse effects with other antidepressants. Notably, trazodone was considered useful in patients with MDD and epilepsy as it has a low impact on seizure threshold (similar for SSRIs) [16].

The panelists agreed that trazodone is suitable for young adults (≥18 years) but the agreement level was 69%, which fell short of the 70% threshold for strong consensus. Several factors may explain why only moderate consensus was achieved for this group. Owing to the specific properties of trazodone, clinicians are more used to prescribing it to mature adults and the elderly and have less experience with younger adults. The lack of licensing for children and adolescents, combined with limited robust safety data for young adults, may have caused some experts to exercise caution regarding trazodone use in this population. Also, the panelists may have been concerned about age-related vulnerabilities owing to incomplete neurodevelopment, and about increased sensitivity to sedation and orthostatic hypotension risk. Moreover, regulatory warnings that antidepressant use in individuals under 25 years of age with psychiatric disorders may increase the risk of suicidality could have reduced panelists’ willingness to recommend trazodone for this age group, despite its clinical benefits.

Moderate consensus of agreement was also reached for patients with MDD and bipolar disorder, emotional blunting, hypertension, PTSD, or migraine. The lack of a strong consensus for these patient profiles may also be due to insufficient data. In cases such as MDD with migraine or emotional blunting, the availability of evidence for other medications (e.g., amitriptyline for migraine in Italy), but not trazodone, may have contributed to the limited agreement. Despite only a moderate consensus in this study, trazodone use in cases of MDD with PTSD or bipolar disorder is common, in particular to treat symptoms such as anxiety and sleep disturbances that occur in those conditions. Similarly, hypertension is not a contraindication for the use of trazodone, but the lack of a strong consensus may have been impacted by a lack of experience in treating these patients.

The strong neutral consensus (i.e., neither agreement nor disagreement) for patients with MDD and QT prolongation or arrhythmia may be a reflection that each patient in these categories needs to be evaluated depending on their individual characteristics.

Among the 14 statements with no consensus, 5 statements approached the agreement threshold with concurrence among more than 50% of physicians. These included whether trazodone should be used in patients with MDD associated with eating disorders (58% agreed; 6% disagreed) and whether trazodone should be used in patients with MDD and anhedonia symptoms (59% agreed; 6% disagreed), chronic kidney disease (58% agreed; none disagreed), atypical depression subtype (53% agreed; 6% disagreed), or OCD (52% agreed; 6% disagreed). One statement – whether trazodone should be used in patients with MDD and risk of priapism – had disagreement among more than 50% of physicians (59% disagreed; 28% agreed).

Trazodone is often primarily perceived as an antidepressant with hypnotic properties, which may lead to its potential benefits on anhedonia being underestimated, despite some supporting evidence [17, 18]. In a study of 79 patients with MDD, for example, anhedonia has been shown to be improved by both first- and second-line treatment with extended-release trazodone [18]. However, we cannot rule out the possibility that improvements in anhedonia are an indirect effect secondary to improving sleep, mood and anxiety.

While evidence on trazodone use in patients with MDD and comorbid eating disorders is limited, it is considered to have a weight-neutral profile, which may represent an advantage in patients with MDD and obesity. In patients with bulimia nervosa, trazodone has been reported to reduce the frequency and urgency of binge eating episodes [19, 20]. Trazodone is generally well tolerated in patients with mild-to-moderate chronic kidney disease, with no need for dose adjustment in most cases, although caution should be used in patients with severe renal impairment [6].

Panelists identified patients with MDD and sleep disturbances, anxiety or agitation as those who would benefit most from treatment with trazodone. Effectiveness in these conditions may also explain trazodone’s effectiveness in patients with comorbid MDD and alcohol use disorders [21]. Elderly patients, in whom trazodone is commonly used as add-on treatment, also ranked highly among those most likely to benefit from trazodone.

### Study limitations

This study has a number of limitations. First, there was a low number of participants, which, while sufficient for a Delphi process, may not capture the full range of perspectives across Europe. Second, panelists and SC members were selected based on clinical and academic experience. While this selection ensures an expert perspective for the Delphi process due to the participants’ extensive knowledge, it may introduce selection bias and limit the generalizability of the findings. The majority of panelists were psychiatrists, with only a small percentage of general neurologists, which may skew the perspective towards psychiatric practice; in addition, the process did not include input from general practitioners, other healthcare providers, or patients. Furthermore, while participants came from eight European countries, representation was not always proportional and some countries may be less represented than others. Consensus statements were shaped by the current level of evidence, which in some areas remains limited. Finally, trazodone formulations may vary across countries, which could influence clinical practice and how some statements are interpreted by participants.

In conclusion, this Delphi panel provides expert consensus on the clinical use of trazodone in MDD, highlighting specific patient profiles where its use may be particularly beneficial. The findings offer practical guidance to support more individualized and evidence-informed decisions in clinical practice. The consensus reached may complement existing clinical trials data and help inform both future research and updates to treatment guidelines.

## Supporting information

10.1192/j.eurpsy.2026.10162.sm001 Young et al. supplementary material Young et al. supplementary material

## Data Availability

All data associated with this article is available on request from the authors.

## Supplementary material

The supplementary material for this article can be found at http://doi.org/10.1192/j.eurpsy.2026.10162.

## Author’s statement

We confirm that we have read the Journal’s position on issues involved in ethical publication and affirm that this report is consistent with those guidelines.
