# Supplementary material for: Expert consensus on the use of trazodone in patients with major depressive disorder: results from a European Delphi panel
Source: Eur Psychiatry. 2026 Feb 6;69(1):e36. doi: 10.1192/j.eurpsy.2026.10162 (PMC13122520; doi:10.1192/j.eurpsy.2026.10162)
Supplement: Young et al. supplementary material [file S092493382610162Xsup001.docx]

**Supplementary Figure 1. Literature search**


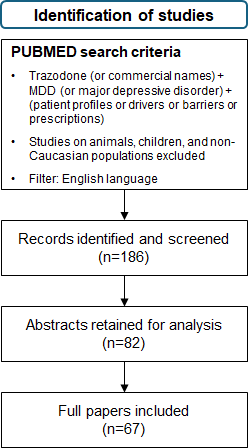


MDD, major depressive disorder.

**Supplementary Table 1. Panelists’ characteristics and experience**

|  | Psychiatrists  (n=27) | Neurologists  (n=5) | All  (N=32) |
| --- | --- | --- | --- |
| Country of practice, n (%)  Austria  Bulgaria  Italy  Poland  Portugal  Romania  Spain  Turkey | 3 (11)  2 (7)  7 (26)  5 (19)  5 (19)  1 (4)  3 (11)  1 (4) | 1 (20)  0 (0)  1 (20)  0 (0)  2 (40)  0 (0)  1 (20)  0 (0) | 4 (13)  2 (6)  8 (25)  5 (16)  7 (22)  1 (3)  4 (13)  1 (3) |
| Years in specialty, years, n (%)  5–10  11–15  >15 | 5 (19)  6 (22)  16 (59) | 0 (0)  0 (0)  5 (100) | 5 (16)  6 (19)  21 (66) |
| Years in MDD, n (%)  5–10  11–15  >15 | 3 (11)  3 (11)  21 (78) | 0 (0)  0 (0)  5 (100) | 5 (16)  1 (3)  26 (81) |
| Experience in specific conditions, n (%)  Cognitive impairment  Chronic pain  BZD withdrawal  Substance abuse withdrawal  Neuropathic pain  Epilepsy | 26 (96)  25 (93)  23 (85)  22 (81)  19 (70)  16 (59) | 5 (100)  4 (80)  4 (80)  2 (40)  4 (80)  2 (60) | 31 (97)  29 (91)  27 (84)  24 (75)  23 (72)  19 (59) |
| Type of practice centre(s), n (%)  University or research hospital  Public hospital  Private hospital  Private centre  Public outpatient clinic  Private outpatient clinic | 21 (78)  5 (19)  2 (7)  3 (11)  5 (19)  11 (41) | 2 (40)  3 (60)  2 (40)  0 (0)  2 (40)  2 (40) | 23 (72)  8 (25)  4 (13)  2 (9)  7 (22)  13 (41) |
| Role in hospital/service, n (%)  Chief of Unit  Department Head  Consultant/Specialist | 8 (30)  7 (26)  12 (44) | 3 (60)  0 (0)  2 (40) | 11 (34)  7 (22)  14 (44) |
| Patient number, median (IQR)  Diagnosed/treated per month (all)  Diagnosed/treated per month (MDD)  Newly diagnosed MDD | 100 (80–150)  50 (30–70)  15 (10–20) | 100 (75–260)  30 (25–35)  4 (0.5–19) |  |
| Involvement in MDD patient journey, n (%)  Diagnosis  Treatment initiation  Treatment adjustment  Long-term monitoring/follow-up  Non-pharmacological treatments | 27 (100)  27 (100)  27 (100)  26 (96)  22 (81) | 5 (100)  5 (100)  4 (80)  5 (100)  1 (20) | 32 (100)  32 (100)  31 (97)  31 (97)  23 (72) |

BZD, benzodiazepine; IQR, interquartile range; MDD, major depressive disorder.
